# *Aedes*–Chikungunya Virus Interaction: Key Role of Vector Midguts Microbiota and Its Saliva in the Host Infection

**DOI:** 10.3389/fmicb.2019.00492

**Published:** 2019-04-09

**Authors:** Valter Vinícius Silva Monteiro, Kely Campos Navegantes-Lima, Alessandra Bittencourt de Lemos, Guilherme Liberato da Silva, Rafaelli de Souza Gomes, Jordano Ferreira Reis, Luiz Carlos Rodrigues Junior, Onilda Santos da Silva, Pedro Roosevelt Torres Romão, Marta Chagas Monteiro

**Affiliations:** ^1^Laboratory of Inflammation and Pain, Department of Pharmacology, Ribeirão Preto Medical School, University of São Paulo, Ribeirão Preto, Brazil; ^2^Graduate Program in Neuroscience and Cellular Biology, Biology Science Institute, Federal University of Pará, Belém, Brazil; ^3^Department of Microbiology, Immunology and Parasitology, Federal University of Rio Grande do Sul, Porto Alegre, Brazil; ^4^Laboratory of Acarology, Tecnovates, University of Taquari Valley – Univates Lajeado, Lajeado, Brazil; ^5^Graduate Program in Pharmaceutical Science, Health Science Institute, Federal University of Pará, Belém, Brazil; ^6^School of Pharmacy, Health Science Institute, Federal University of Pará, Belém, Brazil; ^7^Laboratory of Cellular and Molecular Immunology, Federal University of Health Sciences of Porto Alegre, Porto Alegre, Brazil

**Keywords:** *Aedes aegypti*, *Aedes albopictus*, arthropod-borne virus, CHIKV, virus maintenance, microorganisms

## Abstract

*Aedes* mosquitoes are important vectors for emerging diseases caused by arboviruses, such as chikungunya (CHIKV). These viruses’ main transmitting species are *Aedes aegypti* and *Ae. albopictus*, which are present in tropical and temperate climatic areas all over the globe. Knowledge of vector characteristics is fundamentally important to the understanding of virus transmission. Only female mosquitoes are able to transmit CHIKV to the vertebrate host since they are hematophagous. In addition, mosquito microbiota is fundamentally important to virus infection in the mosquito. Microorganisms are able to modulate viral transmission in the mosquito, such as bacteria of the *Wolbachia* genus, which are capable of preventing viral infection, or protozoans of the *Ascogregarina* species, which are capable of facilitating virus transmission between mosquitoes and larvae. The competence of the mosquito is also important in the transmission of the virus to the vertebrate host, since their saliva has several substances with biological effects, such as immunomodulators and anticoagulants, which are able to modulate the host’s response to the virus, interfering in its pathogenicity and virulence. Understanding the *Aedes* vector-chikungunya interaction is fundamentally important since it can enable the search for new methods of combating the virus’ transmission.

## Introduction

The sylvan transmission cycle involving invertebrate vector(s) and vertebrate reservoirs maintains, amplifies and contributes to arboviruses that actively provoke outbreaks of yellow fever, West Nile, Zika, chikungunya, and dengue (Rodríguez-Morales, 2015; Vega-Rúa et al., 2015; Chouin-Carneiro et al., 2016). CHIKV is a mosquito-borne arthritogenic pathogen, classified as an alphavirus of the Togaviridae family, which has an envelope and single strand RNA as nucleic acids. In the urban cycle, this virus is particularly transmitted by the bites of mosquitoes from the genus *Aedes*, mainly *Aedes aegypti* and *Aedes albopictus*, causing arthritis or arthralgia, which is accompanied by fever and rash (Mourya and Yadav, 2006). In this regard, *Ae. aegypti* is the most important vector and has extensive worldwide distribution, existing mainly in tropical and subtropical areas, whereas *Ae. albopictus* can thrive in temperate regions. Thus, *Aedes* mosquitoes are known to be the primary vectors of emerging and re-emerging arbovirus diseases. Arbovirus disease transmission cycles can be initiated by female mosquito during the blood meal (Honório et al., 2015; Kraemer et al., 2015).

These vectors are adapted for indoor and daytime biting in urban areas. They feed primarily in the indoors and the outdoors near dwellings, and choose standing water, such as puddles and water containers, to procreate (Rodríguez-Morales, 2015). Indeed, their larvae can be found in artificial containers where other microorganisms can be found. Consequently, these mosquito species can harbor many microorganisms, which can affect several physiological functions of the mosquito, such as aiding in digestion, nutrition, and reproduction (Gaio et al., 2011). In fact, the vector mosquito microbiota can modulate its immune response, as well as its ability to eliminate pathogens that cause diseases in humans (Dennison et al., 2014; Hegde et al., 2015).

**FIGURE 1:**
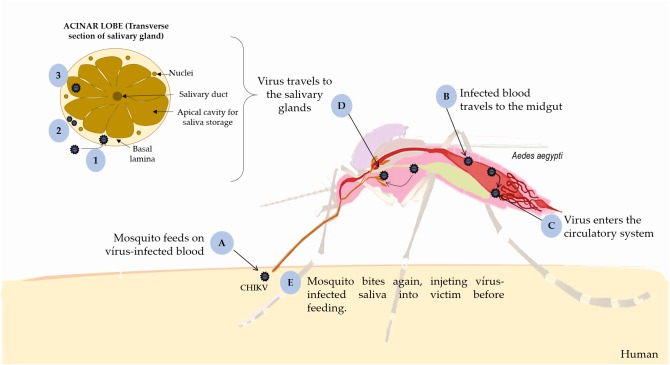
Replication of CHIKV in mosquito. **(A)** the mosquito feeds on virus-infected blood and occurs intrinsic incubation period in the vector**, (B)** CHIKV infects the midgut cells, **(C)** replicates and disseminates to the hemocoel and other organs, such **(D)** salivary glands. **(1)** It penetrates the basal lamina of the salivary glands surrounding acinar cells; **(2)** replicates inside these cells; and **(3)** is deposited into the apical cavities, **(E)** where mosquito saliva is stored prior to its release during feeding. This figure used elements from Servier Medical Art (www.servier.com).

Several studies identify the digestive tract as the first structure to be influenced by microorganisms, since it is the organ that receives and processes the ingested material (Franz et al., 2015). Once in the mosquito midgut, this microbiota is able to interfere with the vector-host-microorganism relationship (Weaver and Reisen, 2010; Dennison et al., 2014). Findings also evidenced by Hegde et al. (2015), who reported that the microbiota may vary according to the site, where an interaction can take place with the same tissue or target protein of arbovirus, with the virus itself, or even both. This microbiota is colocalized with the ingested arboviruses, mainly in the gut of the mosquito, but also in other tissues, such as those of the germline, salivary glands and Malpighian tubules (Dubrulle et al., 2009; Moreira et al., 2009a), as shown in Figure 1.

In terms of pathogens infection, viral transmission via the *Aedes* vector is a complex process dependent on intrinsic factors to the mosquito, such as salivary composition, mosquito survival and virus replication index, and extrinsic factors that includes the climate, accessibility of vertebrate hosts and population and competition of vectors (Coffey et al., 2014). There is evidence that mosquito saliva can lead to vascular homeostasis and attenuate the host’s immune response (Melo et al., 2015), thereby enhancing viremia (McCracken et al., 2014) and viral pathogenicity (Schneider et al., 2004, 2010; Wasserman et al., 2004; Schneider and Higgs, 2008; Le Coupanec et al., 2013).

**FIGURE 2:**
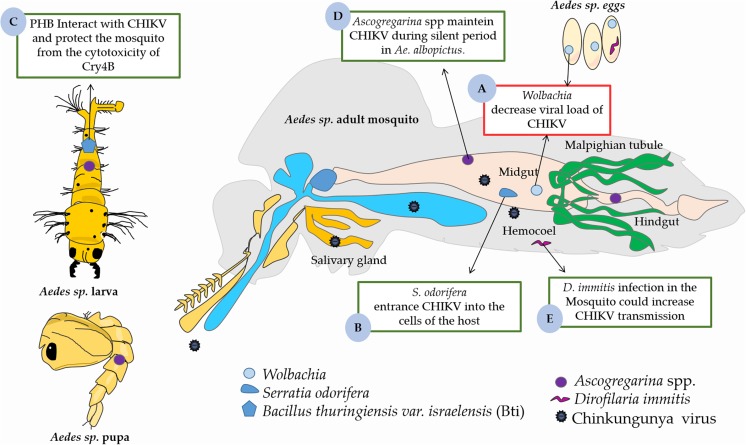
The role midguts microbiota on the CHIKV infection in *Aedes* vector. **(A)** The presence of Wolbachia in midgut decreased viral load in *Aedes* sp. **(B)** *Serratia odorifera* facilitates the entrance of CHIKV into the cells of the host. **(C)** Cry4B of *Bacillus thuringiensis var. israelensis* (Bti) has a direct interaction with PHB (a receptor of CHIKV) of *Aedes sp.*, which can protect the mosquito from the cytotoxicity of Cry4B. **(D)** *Ascogregarina* spp. acts in maintenance of the CHIKV at silent period in *Ae. albopictus*. **(E)** *Dirofilaria immitis* could increasing CHIKV transmission in *Aedes sp.* This figure used elements from Servier Medical Art (www.servier.com).

To develop better prophylactic and therapeutic interventions is essential to know how the immune system works against invading pathogens, especially intracellular microorganisms. Thus, some factors may influence CHIKV infectivity both in the vector and in humans. As such, we will describe some bacteria, protozoa and helminthes that may facilitate the infection and maintenance of CHIKV in *Aedes* mosquitoes, as summarized in Figure 2. In addition, we will also provide a comprehensive overview of the interactions between mosquito saliva and mammalian host immune cells that influence viremia and pathogenicity.

## Overview of the *Aedes* sp. Vector and Virus Interaction

There are over than 3,500 species of mosquitoes widely spread worldwide. They can be further classified into 112 genera, with four genera repeatedly linked with disease transmission to humans in the tropics and in cooler climates: *Aedes, Anopheles, Culex*, and *Ochlerotatus* (Elbers et al., 2015). Therefore, the *Aedes* genus has over 950 species, including *Ae. aegypti* as anthropophilic mosquitoes and *Ae. albopictus* as endophilic mosquitoes, which are more exophilic under natural field conditions; both are the most relevant species in tropical areas regarding disease transmission due to their adaptability to urban life and a high susceptibility to emerging and re-emerging arboviruses (Carrington and Simmons, 2014).

*Aedes aegypti* was first described in Linnaeus (1762), originating in African forests. This species is divided into two subspecies: (i) *Ae. aegypti formosus*, the darker and sylvatic mosquito, which reportedly inhibits forested habitats of Africa and is predominantly zoophilic; (ii) *Ae. aegypti aegypti* is a predominantly anthropophilic and domestic mosquito having as artificial habitats, mainly in urban environments reproducing in standing water containers and is widely diffused in tropical and subtropical areas (Brown et al., 2011; Coffey et al., 2014). *Ae. aegypti* has a bright, silvery, lyre-shaped dorsal pattern, white banded legs and can be found in some Asian countries, Australia, equatorial parts of Africa and in the Americas. More specifically, it can be found in the southern United States, Middle America, and South American countries, and has been highly distributed in Brazil (Kraemer et al., 2015).

*Aedes albopictus* was first described by Skuse (1894) in Calcutta, India. This species has a single, longitudinal, silvery dorsal stripe and white banded legs. It was arose from the forests of southeast Asia, and has been reported in several European countries and some Asian countries; it is poorly distributed in the African continent (Coffey et al., 2014). This species is highly distributed in the southeastern United States and southeast Brazil. In this regard, Brazil is one of the countries where the outbreaks of dengue and CHIKV occurred more frequently (Honório et al., 2015). The possible explanations for these outbreaks were the higher dissemination of both species of *Aedes* in this region and a single E1-A226V mutation (change of an alanine to a valine at codon 226 of the E1 protein) in different CHIKV strains that led specific genetic adaptations in the *Ae. albopictus* vector. Thus, this mutation increased the ability of the virus to replicate in *Ae. albopictus*, favored an increase in virus infectivity in the midgut and its spread to the salivary glands, thereby greatly facilitating the transmission of the disease (Tsetsarkin et al., 2007). Having an overview of the distribution of these species of mosquito vectors, Weaver and Lecuit (2015) also discussed why large epidemic cases of CHIKV have also occurred in Africa and the Indian Ocean basin and are now spreading rapidly throughout Europe and the Americas (Weaver and Lecuit, 2015).

*Aedes aegypti* can share the same larval habitats with *Aedes albopictus*, using natural and artificial water to lay their eggs individually on standing water surfaces because they are not susceptible to desiccation. After the eggs hatch, larvae can develop into four larval stages that metamorphose into pupae, and both are aquatic. After 2 days of the pupa cycle, pupa rupture occurs and the adult mosquito will be fully developed (Bidlingmayer, 1974).

For egg development, female mosquitoes should blood feed to acquire amino acids and other components that are important for the synthesis of vitellogenin, a glycophos apolipoprotein that is released into the insect hemolymph. Then, this protein is carried to the ovaries to be absorbed by the oocytes in the follicular epithelia. The female mosquito can oviposit approximately 250 eggs when the oocytes are full. In general, the place where the oviposition will occur is determinate by temperature, rainfall, relative humidity and wind, and hot and humid climates favor the flight and oviposition of the female mosquito (Bidlingmayer, 1974; Day, 2016). In addition, the main *Ae. aegypti* female behaviors, such as oviposition, blood feeding and sugar feeding, appear to be programmed by an overall circadian activity cycle (Day, 2016). The environment may influence vector-pathogen interactions during larval development, which leads to changes in mosquito competence, distribution and transmission of virus infections including chikungunya, dengue, Zika, and yellow fever to vertebrate hosts. In recent years, several studies from different continents have shown that vector competence can vary between individuals and among mosquito populations, and may be affected by the genetic and the environment components in which the vector is inserted (Diagne et al., 2014; Richard et al., 2016; Agha et al., 2017; Göertz et al., 2017; Chin et al., 2018).

## Chikv–Vector Interactions

Vertical transmission in vectors occurs through infected eggs and horizontal transmission occurs during blood meals on a host, during which female mosquitos ingest CHIKV particles. Initially, CHIKV infects and replicates into midgut cells before disseminate to the hemocele and salivary glands during the extrinsic incubation in the vector (Coffey et al., 2014). Indeed, CHIKV needs reach the basal lamina of acinar cells, replicate inside them and get to the apical side, where mosquito saliva is maintained until releasing during blood meal. In mosquito, the salivary glands are sexually dimorphic, paired set in the thorax, and formed by a distinctive tri-lobed structure (two lateral and one median). These lobes consist of a basal lamina bounded by a singular epithelial layer or acinar cells that produces saliva, around a central salivary duct were the saliva is retained and later injected into the host’s skin during feeding (Juhn et al., 2011; Melo et al., 2015; Vega-Rúa et al., 2015) (Figure 1).

Moreover, during egg oviposition in artificial containers, the mosquitoes share larval habitats with some microorganisms. Consequently, they harbor many microorganisms that can develop and colonize their tissues, especially in the digestive system, where they can affect physiological functions, such as nutrition, digestion, and reproduction (Gaio et al., 2011). In addition, the mosquito microbiota can impact the vector’s competence for human pathogens (Hegde et al., 2015). Several studies identify the digestive tract as the first structure to be influenced by microorganisms, since it is the organ that receives and begins to process ingested material (Franz et al., 2015). Once in the mosquito midgut, this microbiota is able to interfere in the vector-host-microorganism relationship (Weaver and Reisen, 2010; Dennison et al., 2014).

In this regards, Hegde et al. (2015) reported an interaction can take place with the same tissue or target protein of the arbovirus, with the virus itself or even both, depending on the site. In addition, the variability of the microbial community in mosquito seems to be modulated by diet, sex, species and life stage. In the midgut of the mosquito, bacteria, protozoa and also helminthes are often located close to the habitats of ingested arboviruses, however, they can also infect other tissues, such as germline, salivary glands and Malpighian tubules (Dubrulle et al., 2009; Moreira et al., 2009a). In addition, Apte-Deshpande et al. (2014) showed that some of these mosquito gut inhabitants can also influence infections of CHIKV, DENV and yellow fever virus (YFV) in *Aedes* mosquitoes.

## Role of the Midguts Microbiota on the Chikungunya Virus Infection in *Aedes* Vector

### Key Role of Endosymbiotic Bacteria

Several bacteria can often be found in the mosquito gut, as well as in the tissues of the germline, Malpighian tubules and salivary glands (Favia et al., 2007; Hughes and Rasgon, 2014; Sharma et al., 2014; Chavshin et al., 2015). For instance, in *Anopheles culicifacies*, Sharma et al. (2014) found that the salivary gland harbors a broader and more diversified microbiota when compared to the gut of this insect. Moreover, these authors observed a similarity of 11% between the symbiotic bacterial communities of the midgut and the salivary gland of the *Anopheles culicifacies*, which are involved in the digestion of food (Sharma et al., 2014). *Wolbachia* is an obligate intracellular gram-negative endosymbiotic bacterium, which naturally infects more than 60% of insect species, including some *Aedes* species, such as *Ae. albopictus*, but not *Ae. aegypti* (Moreira et al., 2009a). This bacterium can be transmitted between mosquitoes by means of vertical transmission (maternally inherited) or by horizontal (e.g., parasitoid-host transfer), providing to its host nutritional benefits and greater resistance to pathogens (Hedges et al., 2008; Hegde et al., 2015; Wiwatanaratanabutr and Zhang, 2016).

The presence of *Wolbachia*-interfering pathogens, such as virus, was initially observed by Hedges et al. (2008) who showed that *Drosophila melanogaster* that contained *Wolbachia* bacteria in their microbiota had a mortality delay induced by *Drosophila* C virus (DCV), whereas flies without *Wolbachia* were more susceptible to the virus infection (Hedges et al., 2008). Thus, these authors have suggested that the delay in mortality caused by *Wolbachia* is associated with the anti-viral action of this bacterium. Similarly, Teixeira et al. (2008) also observed that the presence of *Wolbachia* decreased viral load in Drosophila, as well as Nora virus, but only slightly Flock House virus (FHV). In addition, these authors reported that genes involved in resistance to DCV (Dcr-2, ago-2, hop and *w*Mel) in *Drosophila* can be stimulated by the presence of *Wolbachia* (Teixeira et al., 2008).

Although, *Wolbachia* does not naturally infect the *Ae. aegypti*, this mosquito can be transfected with the bacterium by embryo microinjection and adult microinjection (Hughes and Rasgon, 2014). In this regards, Moreira et al. (2009a) also showed that *Wolbachia* influences the dispersion of the DCV along the body of the mosquito. First, they used three distinct groups of mosquitoes: one infected with the *w*MelPop-CLA strain of *Wolbachia*, another treated with tetracycline, and a wild control group, free of all bacteria. The insects were fed blood containing a viral particle and the dispersion of this content along the head and body, as well as along the legs and wings, was observed. Moreira et al. (2009a) highlights the difference in the amount of viral RNA copies after the 14th day of infection, where the group infected with *Wolbachia* only 17% of the mosquitoes were infected with CHIKV, yet none of the animals had the virus disseminated through their bodies. The authors concluded that the increasing difficulty of *Ae. aegypti* in completing the process of blood feeding had been related to these strains of *Wolbachia* (Moreira et al., 2009a).

In addition, when associated with the same host, CHIKV and *Wolbachia* seem to interact with each other once values such as density, dispersal and movement of the virus within the mosquito change with the presence of the bacterium, and vice versa (Moreira et al., 2009a). Mosquitoes naturally infected by *Wolbachia* showed some resistance to viruses; however, the resistance seemed less effective (60% for *Wolbachia*++ and 70% for *Wolbachia* -) in comparison with transinfected *Wolbachia* strains such as *w*MelPop-CLA (Moreira et al., 2009b). Therefore, *Wolbachia*-infected (with *w*MelPop strain) *Ae. aegypti* mosquitoes showed reduced vector capacity and blood feeding behavior through physiological changes, such as reduction of females’ fecundity and egg viability and hatch rate, antiviral protection, stimulation of the immune system and decrease in the amount of saliva produced by the vector (Moreira et al., 2009b; Johnson, 2015; Fraser et al., 2017). In this regard, Moreira et al. (2009b) reported that older *Ae. aegypti* females infected with wMelPop had an increase in pre-probing and probing time, as well as exhibiting shaking behavior and the bendy proboscis, produced less volume of saliva and less ability to feed on blood. However, *Wolbachia* infection did not alter the activity of salivary apyrase (Moreira et al., 2009b).

Thus, the presence of the *w*MelPop strain blocks the accumulation of CHIKV virus (Moreira et al., 2009a). However, other studies have shown that the antiviral action of *Wolbachia* strains is associated with the induction of antimicrobial peptides and pre-activation of the innate immune response in the insect (Kambris et al., 2009, 2010; Moreira et al., 2009a; Fraser et al., 2017). For instance, *Ae. aegypti* induced the expression of molecules responsible for the immune effect (cecropin, defensin, thio-ester containing proteins, and C-type lectins) in the presence of *w*MelPop-CLA *Wolbachia* (Moreira et al., 2009a).

In *Ae. albopictus* vector, Zouache et al. (2012) analyzed the impact of CHIKV infection on bacterial community of this insect and found a significant decrease in endosymbiontic bacteria *Wolbachia* and *Blattabacterium*. In contrast, the authors showed an augment in bacteria from the *Enterobacteriaceae* family, as well as CHIKV infection influenced the composition of the bacterial community, and the aging of these infected mosquitoes changed slightly the density of bacteria, probably due to modified nutritional conditions (Zouache et al., 2012). *Ae. albopictus* is naturally infected by two strains of *Wolbachia*, named *w*AlbA and *w*AlbB (Zouache et al., 2009, 2012), but using C6/36 cell line (from *Ae. albopictus* cells) infected *in vitro* with *w*AlbB strain, it was demonstrated its ability to inhibit the replication and assembly/secretion steps of CHIKV infection (Raquin et al., 2015). Nevertheless, in some cells CHIKV RNA and *Wolbachia* were co-localized (Raquin et al., 2015).

Mousson et al. (2010) also studied the modulation of CHIKV replication in *Ae. albopictus* (Mousson et al., 2010). Using RT-PCR techniques to determine the viral load through RNA and PCR, these authors determined the density of *Wolbachia* by the presence of the actin gene in the DNA. However, mosquitoes with *Wolbachia* showed a homogeneous pattern in the amount of virus, associated with a decrease in the bacterial load. The authors stated that this had probably occurred due to competition for resources within the host.

van den Hurk et al. (2012) study the influence of *w*Mel strain of *Wolbachia* on CHIKV infection in *Ae. aegypti* and showed that CHIKV was detected significantly less often in mosquito bodies infected with the *w*Mel strain (23%), compared to uninfected mosquitoes (83%), and decreased dissemination to the salivary glands (van den Hurk et al., 2012). Regardless of all these studies, differently from *Ae. albopictus* that is naturally infected by *w*AlbA and *w*AlbB strains of *Wolbachia*, wild *Ae. aegypti* did not carry this bacterium (Zouache et al., 2009, 2012; Hughes and Rasgon, 2014). Although, recently, some studies have detected *Wolbachia* in a natural population of *Ae. aegypti* (Coon et al., 2016; Goindin et al., 2018).

Some species of *Serratia* (Enterobacteriaceae) have been isolated from the gut microbiota of mosquitoes, where they were considered to be dominant in all isolation assays. They represented more than half of the total microorganisms (fungi and bacteria) in *Ae. aegypti* (Demaio et al., 1996; Gusmão et al., 2007). *Serratia* spp. may possess some competitive advantages over other bacteria (Gusmão et al., 2007), thereby *Serratia marcescens* and *Serratia nematodiphila* are investigated for the control of some mosquito species (Patil et al., 2011, 2012; Suryawanshi et al., 2015).

Initially, Arnoult et al. (2009) suggested the mitochondrial proteins are important to the immune response of many organisms, since they act on the induction of cellular death after an infection. In this way, the authors suggest that it is possible that Gram-negative *Serratia odorifera* is a facilitator of the entrance of CHIKV into the cells of the host, which would increase the success of viral infection in the vector (Arnoult et al., 2009).

Apte-Deshpande et al. (2014) fed *Ae. aegypti* females, free of midgut microbiota, with virus alone, *S. odorifera* or *Microbacterium oxydans* along with virus via blood meal. These three groups were monitored for dissemination of CHIKV (Apte-Deshpande et al., 2014). The mosquitoes that were fed with *S. odorifera* and virus presented a higher susceptibility to CHIKV compared to the groups that received only virus or *M. oxydans*. On day 10 post feeding (DPF), the group receiving CHIKV+*S. odorifera* in the blood meal showed slightly higher virus titers (7.563 × 10^7^ pfu/mosquito) compared to groups receiving only virus (4.93 × 10^7^ pfu/mosquito) or virus plus *M. oxydans* (4.15 × 10^7^ pfu/mosquito), indicating enhanced replication of the virus in *Ae. aegypti* tissues. In addition, these authors also showed that increased susceptibility of *Ae. aegypti* to CHIKV mediated by *S. odorifera*, is due to interaction of the P40 protein of the bacterium with proteins prohibitin and porin present on the midgut of *Ae. aegypti* that immunosuppress the mosquito, thus accentuating the infection to CHIKV (Apte-Deshpande et al., 2014). Thus, as the virus replication is not even two-fold elevated might be no effect on virus particles due to co-infection (Apte-Deshpande et al., 2014).

Another very important specie of bacteria is *Bacillus thuringiensis var. israelensis* (Bti), a gram-positive entomopathogenic bacterium that has been widely used in the control of mosquitoes (Boyce et al., 2013). This bacteria’s efficacy can be due to the action of six proteins (Cry4Aa, Cry4Ba, Cry10Aa, Cry11Aa, Cyt1Aa, and Cyt2Ba) (Berry et al., 2002; Park et al., 2005), which are deposited in inclusions that become part of the parasporal crystal of Bti (Bayyareddy et al., 2009). The main mechanism of toxicity of Cry proteins is the lysis of midgut epithelial cells mediated by the formation of pores (Bravo et al., 2007). Cry4B is one of the most effective toxin against *Aedes* mosquitoes. In this sense, it was reported that target proteins in the midgut of *Ae. aegypti* larvae, including the prohibitin (PHB), which may also be a receptor for the entry of DENV into mosquito cells (Bayyareddy et al., 2009; Kuadkitkan et al., 2012), as well as it may also be target of the mosquitocidal Cry4B protein. Kuadkitkan et al. (2012) have shown that *Aedes* cell lines bound to PHB are more susceptible to DENV infection, however, they do not undergo cytolysis in the presence of the Cry4B toxin (Kuadkitkan et al., 2012). These authors also showed a considerable colocalization between Cry4B and PHB in whole cells, which can protect the mosquito from cell death (Kuadkitkan et al., 2012). Therefore, Wintachai et al. (2012) were the first to identify that PHB is also a CHIKV receptor protein in the mosquito gut. Thus, we can suggest that PHB has a direct interaction with CHIKV and Cry4B during infection, which can protect the mosquito from the cytotoxicity of Cry4B (Wintachai et al., 2012).

### Key Role of Protozoans

In *Ae. aegypti* and *Ae. albopictus* has been reported high prevalence of some species of *Ascogregarina* (Apicomplexa: Lecudinidae), such as *Ascogregarina culicis* and *Ascogregarina taiwanensis*, respectively (Blackmore et al., 1995). Larvae become infected after ingestion of oocysts containing sporozoites from its habitat and are vulnerable to gregarine infection at all larval instars (Kobayashi, 2006; Erthal et al., 2012) The sporozoites of *Ascogregarina* infects the epithelial cells of mosquitoes, develop the intracellular form (trophozoite) in the midgut, and subsequently rupture the epithelial cells and are released into the intestinal lumen (Chen, 1999; Lantova and Volf, 2014). Muniaraj et al. (2010) related the role of the cyst of *Ascogregarina* in maintenance of the CHIKV during silent period in *Ae. albopictus*. As the midgut is the primary region for virus replication in the mosquitoes, Mourya et al. (2003) stated that CHIKV may exploit these parasites for its own survival and transmission, being maintained in the oocysts of the parasites for a some period of time in nature. After filling breeding places with water, a new mosquito cycle starts and emerging larvae pick up the infection of the parasite by ingesting the oocysts that harbor the virus. So, the emerging adults start the mosquito-human-mosquito virus transmission cycle (Mourya et al., 2003).

Mourya et al. (2003) suggest that the gregarine *A. culicis* could help to maintain the CHIKV inside the mosquitoes. They demonstrated that uninfected first instar *Ae. aegypti* larvae fed on the dried adult mosquito homogenates obtained from the larvae that were exposed to oocysts, and CHIKV extracts were positive for viral antigens (Mourya et al., 2003). However, the uninfected larvae that fed only on the dried virus stock were negative, indicating that the virus does not survive in dried conditions. Therefore, the authors suggested that *Ascogregarina* spp. might play a crucial role in the maintenance of CHIKV during the inter-epidemic phases. Later, Muniaraj et al. (2010) described severe outbreaks of chikungunya fever during 2006–2007 in Kerala and Tamil Nadu, India (Muniaraj et al., 2010). Based on the hypotheses of Mourya the authors investigate the prevalence of *Ascogregarina* spp. in *Ae. albopictus* in 35 samples from different containers. They found a prevalence of 71.62% of *Ascogregarina* infection (including larvae, pupae and adults). If *Ascogregarina* could really be responsible for *Aedes* mosquito’s infection, as suggested by both authors, a more precise study must be conducted (Muniaraj et al., 2010).

### Key Role of Nemathelminthes

To date, just few studies relating Nemathelminthes and Chikungunya virus transmissions have been conducted (Zytoon et al., 1993a,b). Zytoon et al. (1993b) described that *Ae. albopictus* infected with microfilariae of *Dirofilaria immitis* were more susceptible to transovarial transmission of CHIKV whereas non-infected mosquitoes did not transmit the CHIKV to the F1 progenitor. They feed the mosquiotes with defibrinated sheep blood containing 5 × 10^7^ PFU of CHIKV and 20,000 microfilariae of *D. immitis* per milliliter observing that eggs of the first ovarian cycle were infected with CHIKV, which indicates that transovarial transmission occurred following coinfection by dissemination of the virus from female mosquitoes’ ovarian cells to eggs during fertilization (Zytoon et al., 1993b). In addition, the group also described that the microfilariae movement through the gut wall of *Ae. albopictus* increased CHIKV dissemination in the mosquito allowing some virions attached to the sheath of microfilariae to be carried to the hemocoel or that virus particles could leak through the holes produced when the parasite penetrate the gut wall and move into the hemocoel (Zytoon et al., 1993a).

In addition, Montarsi et al. (2015) described that *Aedes koreicus*, a new invasive species of mosquito in Europe, is a competent vector of *D. immitis*. The microfilariae was found in many parts of this mosquito (Montarsi et al., 2015). Later, Ciocchetta et al. (2018) explored the potential of *Ae. koreicus* to transmit CHIKV (Ciocchetta et al., 2018). They observed the dissemination of the virus to the wings and legs of the mosquito and infection of mosquito saliva, with live virus, occurred in two mosquitoes. These data demonstrate that *Ae. koreicus* could be a possible vector of CHIKV transmission in Europe (Ciocchetta et al., 2018). Those data corroborating with the fact that the mosquito can infected with *D. immitis*, could increasing the possibility of virus transmission. However, more studies have to be conducted in order to confirm the competence of this vector for CHIKV.

## Chikungunya Virus Infection in Vertebrate Hosts

CHIKV was first discovered in 1952 in Tanzania. Its name is came from the Makonde word, and describes the body movement stiffness associated with persistent arthralgic symptoms presented by the host, after virus infection. Currently, chikungunya fever is a global epidemic, affecting millions of people due to several factors, including an increased number of travelers and the geographic distribution of mosquito vectors around the world (Mourya et al., 2003; Mourya and Yadav, 2006; Tsetsarkin et al., 2007). Symptoms of CHIKV include high fever, headache, rigors, photophobia and a petechial or maculopapular rash which is sometimes associated with arthralgia and myalgia (Schwartz and Albert, 2010; van Duijl-Richter et al., 2015). Some CHIKV-infected individuals present more severe symptoms, including hemorrhage, hepatitis, cranial nerve palsies and/or Guillain-Barre syndrome (Petitdemange et al., 2015; Mehta et al., 2018).

CHIKV belongs to the *Togaviridae* family, along with 29 different species of the *Alphavirus* genus; they cause diseases in humans and other mammals. The virus is small, with a 60–70 nm diameter, enveloped, spherical, positive-strand RNA virus with genomes of approximately 12 kb, encoding two polyproteins (Solignat et al., 2009; Hollidge et al., 2011). The first polyprotein consists of four non-structural units (nsP1, nsP2, nsP3, and nsP4) and the second is a structural polyprotein composed of five expression units (Capsid, E3, E2, 6K and E1). A sub genomic positive-strand RNA denominate 26SRNA is replicated from a negative-stranded RNA intermediate, which serves as template for the synthesis of viral structural proteins. A subgenomic positive-strand RNA denominate 26SRNA is transcribed from a negative-stranded RNA intermediate, which serves as template for the synthesis of viral structural proteins. Most of the alphaviruses have conserved domains involved in regulation of viral RNA synthesis. The E1 and E2 glycoproteins can form heterodimers that cover the viral surface uniformly and modulates the attachment of the virus to the host cells (van Duijl-Richter et al., 2015). The control of viral replication is regulated exogenous RNAi pathway in mosquitoes; this could limit potential pathologic effects and facilitate the arthropod survival (Coffey et al., 2014).

The infection starts when an infected mosquito bites the susceptible human host. The CHIKV spreads through the dermis infecting fibroblasts, macrophages and monocytes following an acute viremia that reaches 10^9^–10^12^ viral particles per milliliter (Her et al., 2010; Wikan et al., 2012). The receptors used by CHIKV are ubiquitously expressed in many cells, specifically prohibitin, phosphatidylserine, glycosaminoglycans and ATP-synthase β subunit receptors that have been previously characterized (van Duijl-Richter et al., 2015). Interestingly, PBMCs are not the main source of the virus upon infection, suggesting that dermal fibroblasts, endothelial cells and monocytes or macrophages keep reproducing the virus during the infection progress (Sourisseau et al., 2007; Kumar et al., 2012). Since most of these target cells reside in the peripheral tissues, this could be the reason why the CHIKV viremia decreases significantly about 7 days after infection, even with the virus remaining in the body. Chronic disease is associated with the viral persistence in muscle and synovial cells and is manifested by myalgia and arthralgia, which can persist for years (Burt et al., 2017).

Several studies have already reported that CHIKV replicates along the skin before reaches the bones and liver (Schwartz and Albert, 2010; Lo Presti et al., 2014). *In vitro* experiments demonstrated that CHIKV can replicates in human epithelial and endothelial cell lines as well as macrophages and fibroblasts (Sourisseau et al., 2007). Patients with acute CHIKV (5–10 days after the onset of symptoms), when compared to a control group, presented higher systemic levels of IL-2R, IL-6, IL-1RA, IL-8, IL-10, MCP-1, IP-10, and MIG, and low levels of RANTES, whereas those in the chronic phase presented higher levels of MCP-1, IL-1RA, IL-6, IL-8, MIP-1α and MCP-1, returning to levels of uninfected controls in the recovered phase (Chaaithanya et al., 2011). IL-6, IL-8, MCP-1 and MIP-1α, MIP-1β have a determinant role in evolution for chronic disease.

Although very rare, neurological manifestations of CHIKV infections have been fully reviewed elsewhere (Brizzi, 2017; Mehta et al., 2018); these are mainly encephalopathy and encephalitis, encephalomyelopathy, myeloneuropathy, Guillain-Barré syndrome and neuro-ocular manifestations in adults and children infected directly via mosquito bites. Neonatal neurological features from vertical transmission also seem to play an important role in vertebrate infections, as has been previous described and reviewed elsewhere (Contopoulos-Ioannidis et al., 2018).

The molecular targets for CHIKV have already been exhaustively reviewed (Bautista-Reyes et al., 2017; da Silva-Júnior et al., 2017). nSP1, nSP2, and nSP3 are important potent inhibitors of the virus, as well as inhibitors to the known E1-E2. This has led to the compilation of a list of all-important molecular targets, as well as new lead molecules, including synthetic and natural products and designed compounds.

## The Immunomodulatory Role of *Aedes* sp. Saliva on the CHIKV Infection in the Human

Many studies show the effects of *Ae. aegypti* insect salivary components and their role in pathogen infections (Styer et al., 2011; Surasombatpattana et al., 2012; Ockenfels et al., 2014). Recently, our study shows that SGE of *Ae. aegypti* was can able improve the survival of murine polymicrobial sepsis modulating neutrophil influx and increasing antioxidant defenses (de Souza Gomes et al., 2018). However, the most studies reported that components of mosquito or tick saliva facilitate the pathogenesis of the virus, improving its replication (Schneider and Higgs, 2008). The mosquito salivary gland produces and secretes molecules (e.g., NADH ubiquinone glutathione s-transferase, animal heme peroxidase, thioredoxin, cytochrome c oxidase) that aid in the digestion of sugars and nectars for their food (Ribeiro and Arca, 2009; Ribeiro et al., 2010); this may affect vascular constriction, blood coagulation, platelet aggregation (Schneider and Higgs, 2008; Ribeiro and Arca, 2009; Ribeiro et al., 2010), D7 protein (Calvo et al., 2009; Ribeiro et al., 2016) and Sialokinin, which modulates host immunity (Schneider et al., 2004; Wichit et al., 2016).

The *Ae. aegypti* salivary gland components modify the local microenvironment to favor the arbovirus infection (Dhawan et al., 2017). Several studies report that mosquitoes infected with CHIKV have an altered composition of salivary gland proteins, such as: putative inosine-uridine preferring nucleoside hydrolase, 30 kDa allergen-like protein, serpin, and angiopoietin-like protein variants (Tchankouo-Nguetcheu et al., 2012; Shrinet, 2018). SRPN26, protein disulfide-isomerase, tubulin beta chain, malic enzyme and RAN are down-regulated in the presence of CHIKV (Shrinet, 2018).

Some of the proteins that are up regulated with immunomodulatory effects have role in the transmission of CHIKV. For example, some proteins found in *Aedes* saliva have similarity to nucleoside hydrolases of other insects, such as the putative inosine-uridine preferring nucleoside hydrolase enzyme; which catalyzes preferentially the hydrolysis of inosine and uridine, and is increased in salivary gland of female mosquitoes, playing a role in blood feeding the insect (Ribeiro and Valenzuela, 2003). Mosquito salivary nucleosidases together with adenosine deaminases (ADA) found in *Ae. aegypti* saliva prevent mast cell degranulation by adenosine converting into hypoxanthine, which inhibits also production of inflammatory cytokines, prostanoids and leukotriene C4. Besides that, ADA produces inosine, which inhibits the production of inflammatory cytokines, decreasing the host response during blood feeding (Ribeiro and Valenzuela, 2003; Ribeiro et al., 2007; Tchankouo-Nguetcheu et al., 2012).

The 30 kDa allergen-like protein (Aed a 3), also called Aegyptin, that inhibits adhesion and aggregation platelet (Chagas et al., 2014). This protein recognizes the specific binding sites integrin a2β1, glycoprotein IV, and Von Willebrand factor (Armiyanti et al., 2015; Peng et al., 2015). The low molecular form (41 kDa) of serpin (serine proteinase inhibitors) is another protein that is up-regulated in *Ae. aegypti* salivary glands infected by CHIKV (Tchankouo-Nguetcheu et al., 2012). Serpin inhibit endogenous proteases, such as the serine proteases involved in regulating coagulation like factor Xa and thrombin (Wasinpiyamongkol et al., 2010). In this regards Serpin facilitates the blood feeding of the vector and as consequence enhance pathogen transmission from vector to host (Oktarianti et al., 2015).

The immunomodulatory effect of *Ae. aegypti* saliva in viral transmission involves local induction of chemotaxis and Th2 polarization bias Th1 (Boppana et al., 2009). Beside these proteins being up regulated in CHIKV, the *Ae. aegypti* salivary gland demonstrates capacity to modulate Th1/Th2 cytokine (Schneider et al., 2004). A study conducted by Thangamani et al. (2010) observed differences in immunological responses of CHIKV infection by needle inoculation compared to by mosquito bite. The infection with the presence of a mosquito’s saliva modulates a Th2 response with IL-4and IL-10 up-regulation (Venugopalan et al., 2014). In addition, the Th1 profile was also down regulated after saliva exposure, reducing IL-2 and IFN-γ levels, and the expression of TLR-3. Moreover, infected and non-infected mosquito bites reduced the expression of Toll-like receptors. The T-cell class shifts to a Th2 phenotype in the first 6 h after inoculation and a high number of eosinophils influx in the area of injection (Thangamani et al., 2010).

On the other hand, the proliferation of naïve lymphocytes is strongly impaired by the presence of SGE. Curiously, antigen-experienced lymphocytes were less sensitive to SGE inhibition, a mechanism dependent on antiapoptotic molecules. Indeed, the suppressive effect of *Ae. aegypti* on lymphocyte proliferation and biology was previously shown, and specific to T CD4 and T CD8 cells and not to DCs (Niedzwiecki et al., 2013).

*Aedes aegypti* saliva acts on the modulation of other immune system cells in favor of CHIKV infection. However, it has not yet been well established which components could be involved in this infection. Recently, Wichit et al. (2017) showed that *Ae. aegypti* saliva facilitates the replication of CHIKV leading to an increasing number of virus in human skin fibroblasts, besides that it also reduces the IFN-inducible gene in CHIKV-infected cells via the JAK-STAT. Among the reservoirs for viral propagation, the saliva of *Ae. aegypti* induces neutrophils the bite site by a mechanism dependent on the production of CXCL2, with the subsequent secretion of IL-1β and CCL-2 that leads to accumulation of neutrophils by directly mobilizing myeloid cells (Pingen et al., 2016).

**FIGURE 3:**
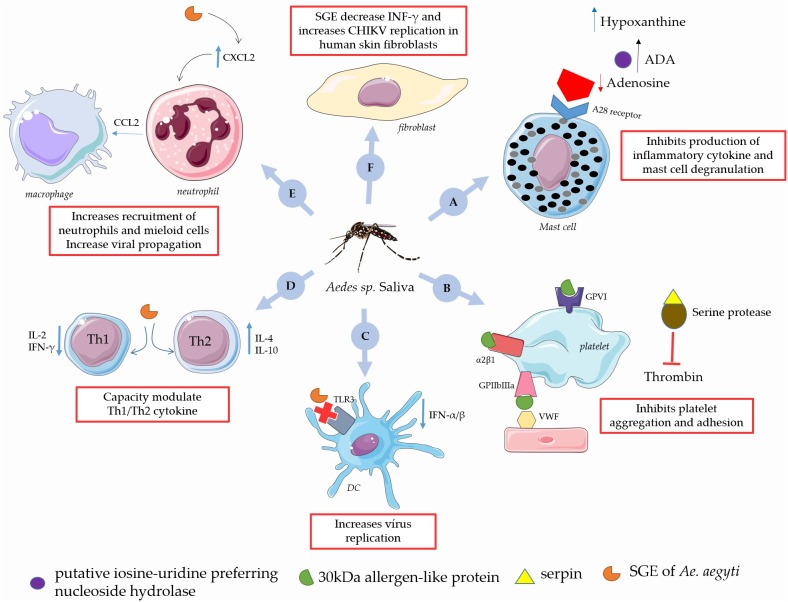
Immunomodulatory role of *Aedes* sp. saliva on the CHIKV infection in the human. **(A)** *Ae. aegypti* salivary putative inosine-uridine preferring nucleoside hydrolase together with adenosine deaminases (ADA) converts adenosine into hypoxanthine, thus preventing production of inflammatory cytokine and mast cell degranulation. **(B)** The 30 kDa allergen-like protein, is a collagen-binding protein that inhibits platelet aggregation, adhesion and serpin inhibit endogenous proteases which is important in the regulating of coagulation. **(C)** SGE of *Aedes* sp. reduce expression of TLR3 and decrease levels of IFN-α/β increasing virus replication. **(D)** SGE of *Ae. aegypti* modulates Th1/Th2 response in favor of CHIKV infection. **(E)** *Ae. aegypti* saliva increases influx of neutrophils to the local bite by a CXCL2 production, with the subsequent accumulated neutrophils and mobilize myeloid cells, which serve as reservoirs for viral propagation**. (F)** SGE of *Ae. Aegypti* increases CHIKV replication in human skin fibroblasts, and down-regulates the interferon IFN-inducible gene in CHIKV-infected cells via the JAK-STAT. This figure used elements from Servier Medical Art (www.servier.com).

In addition, components of the immunomodulatory process of the miRNAs present in *Aedes* saliva can modulate CHIKV infection. Maharaj et al. (2015) tested different types of miRNAs present in saliva of CHIKV infected mosquitoes, including miR-184, miR-12, miR-375 miR-125 and miR-2490. In this study, these miRNAs were silenced to evaluate their importance on CHIKV replication. Silencing these miRNAs promoted a decrease in viral replication rate, suggesting that the presence of these miRNAs up-regulates CHIKV replication or the activation of immune system cells (Maharaj et al., 2015), as shown in Figure 3.

## Conclusion and Perspectives

Arbovirus infections, such as CHIKV, have been growing worldwide and are becoming a major public health problem, especially in immuno-compromised classes such as children and the elderly. CHIKV is dangerous since it can cause arthritis, fever and other impairments, making it necessary to seek new ways to counter the proliferation of this virus. Knowing the vector’s competence is fundamentally important, including an understanding of the vector virus interaction and the modulation of this interaction. Despite advances in the knowledge of mosquito competence in the transmission of CHIKV, little is known with respect to viral infection in the mosquito. However, it is important to highlight that the competence of the mosquito vector can be influenced by several characteristics, including the age of the mosquito, its nutritional status, stage of the gonotrophic cycle, environmental temperature conditions, salivary components and composition of its microbiota (Carrington et al., 2013; Bartholomay and Michel, 2018; Knecht et al., 2018). Thus, investigating the role of local mosquito populations, as well as kinetic and phenotypic characteristics of the different vectors for the transmission of pathogens, is very important in reducing the risk of possible disease outbreaks caused by many viruses, such as zika virus, dengue fever and yellow fever. In this regard, the mosquito microbiota is of fundamental importance to the viral infection of the mosquito. Bacteria of the *Wolbachia* genus are an important advance in the understanding of this interaction, since mosquitoes infected by this bacterium will not present viral infections, thus serving as an important tool in combating these viruses and reducing the transmission of DENV in endemic areas (O’Neill et al., 2018). However, we emphasize that there is a need for more robust work with natural populations, since most studies show the antiviral effects of *Wolbachia* against arboviruses, including DENV and CHIKV, in a model of stable transfection of this bacterium into heterologous mosquito hosts. In addition, mosquito saliva is an important factor in the transmission of CHIKV due to the existence of a series of substances with immunomodulatory and anticoagulants actions. These components may help the mosquito in blood ingestion, but will also allow CHIKV to escape from the host, and are thus fundamentally important to the virulence and pathogenicity of this virus. However, as the microbiota and salivary components of arthropods are highly diverse, together, these factors seem to contribute to the transmission of pathogens; when tested alone, they sometimes oppose the infection and in other situations may facilitate it. Therefore, it is necessary to actively search for new ways to modulate the transmission of CHIKV in the mosquito, which would create a tool to combat the virus.

## Author Contributions

All authors participated in the design of the study and drafted the manuscript. MM participated in the study coordination and helped to draft the manuscript. KN-L have designed and prepared the manuscript figures. All authors read and approved the final manuscript.

## Conflict of Interest Statement

The authors declare that the research was conducted in the absence of any commercial or financial relationships that could be construed as a potential conflict of interest.

## Abbreviations

BsB: binary protein
CHIKV: chikungunya virus
CXCL2: chemokine (C-X-C motif) ligand 2
DC: dendritic cells
DENV: dengue virus
IFN: interferon
Ig: immunoglobulin
IL: interleukin
MCP: monocyte chemotactic protein
MIG: monokine induce by interferon-γ
MIP: macrophage inflammatory protein
NK: natural killer
PBMCs: peripheral blood mononuclear cells
SGE: salivary gland extract
Th: T helper
TNF: tumor necrosis factor
